# Development of Carbon Nanotube Yarn Supercapacitors and Energy Storage for Integrated Structural Health Monitoring

**DOI:** 10.3390/en16155736

**Published:** 2023-08-01

**Authors:** Abdulrahman S. Binfaris, Alexander G. Zestos, Jandro L. Abot

**Affiliations:** 1 Department of Mechanical Engineering, The Catholic University of America, Washington, DC 20064, USA;; 2 Department of Chemistry, American University, Washington, DC 20016, USA;

**Keywords:** carbon nanotube yarn, supercapacitors, energy storage, capacitance, energy density, power density

## Abstract

Developing efficient, sustainable, and high-performance energy storage systems is essential for advancing various industries, including integrated structural health monitoring. Carbon nanotube yarn (CNTY) supercapacitors have the potential to be an excellent solution for this purpose because they offer unique material properties such as high capacitance, electrical conductivity, and energy and power densities. The scope of the study included fabricating supercapacitors using various materials and characterizing them to determine the capacitive properties, energy, and power densities. Experimental studies were conducted to investigate the energy density and power density behavior of CNTYs embedded in various electrochemical-active matrices to monitor the matrices’ power process and the CNTY supercapacitors’ life-cyclic response. The results showed that the CNTY supercapacitors displayed excellent capacitive behavior, with nearly rectangular CV curves across a range of scan rates. The energy density and power density of the supercapacitors fluctuated between a minimum of 3.89 Wh/kg and 8 W/kg while the maximum was between 6.46 Wh/kg and 13.20 W/kg. These CNTY supercapacitors are being tailored to power CNTY sensors integrated into a variety of structures that could monitor damage, strain, temperature, and others.

## 1. Introduction

Different types of energy storage have been developed, including lead–acid, nickel–cadmium, lithium–ion batteries, and ultracapacitors. These can be charged and discharged multiple times without deteriorating [1,2]. Lithium–ion batteries dominate rechargeable batteries in portable devices and electric vehicles because of their exceptional energy density and enduring lifespan [1]. Supercapacitors are electrochemical capacitors with a power density of 104–105 W/kg and an operational life exceeding 1 million cycles [2]. Energy storage is crucial today, supporting countless innovations essential to our daily experiences [3]. Constant energy modules and solid-phase power reservoirs offer potential solutions for energy storage at a high level [4,5]. Flow batteries are an encouraging innovation for grid-scale energy storage owing to their expandability, security, and long-cycle existence. Solid-state batteries utilize a solid electrolyte rather than a liquid one, potentially enabling safer functioning and increased energy compactness [4,5]. Ultracapacitors are a unique energy storage system that fills the gap between standard capacitors and rechargeable batteries. They have high power density and excellent cyclability [6]. Electric double-layer capacitors (EDLCs) accumulate energy through electrostatic charge buildup, which aids their considerable power density and unparalleled endurance [7]. Supercapacitors face considerable obstacles due to their insufficient capacity to store energy relative to batteries. However, promising advancements and uncharted configurations are underway to alleviate these inadequacies [8]. Two significant events were instrumental in advancing carbon nanotube yarns (CNTYs). The first event entails discovering CNT’s existence in 1991 by Iijima [9–12]. Ijima inadvertently chanced upon an entity with empty structures while examining soot, and since then, numerous investigations have been conducted into CNT properties. These inquiries have illuminated emerging utilization opportunities for CNTs involving sensors [13–23]. CNTYsconsist of a CNT assembly for storing energy, especially in supercapacitors. They offer outstanding physical durability, elevated electrical transmittance, and a large area for charge storage. The power density, charge and discharge rate (within seconds), and endurance for over 50,000 cycles could be upscaled to power vehicles, spinn wind blades, ensure planes have backup power, or store wind power for local grids [24]. Two mechanisms enable its capacitance. One is electrochemical double layers where energy is stowed at electrode–electrolyte interfaces. The energy density had electrodes that needed a vast area. Activated carbon has a surface area range of 1000–3000 m^2^/g. Carbon nanotube yarn offers 200–1300 m^2^/g depending on the number of shells. Single-walled nanotubes eclipsed multi-walled ones. The other mechanism is pseudo capacitance from metal oxides’ redox reactions, oxygen or nitrogen groups on carbon, or conductive polymers. Two mechanisms enable its capacitance: electrochemical double layers and a vast area of electrodes. The consequent symmetrical CNTY supercapacitor has a very high specific mass capacitance (208.7–421 F/g), and high areal specific capacitance (3.12–78.3 mF/cm^2^) for the area range and stable cycling (99% capacitance retention following 5000 cycles) [24]. Moreover, the supercapacitor is pliable, and its performance is only lessened by 5% after 100 bending cycles when woven into a glove [24,25]. This study aims to explore the potential of CNTYs in supercapacitor applications. Various materials and configurations were considered for the supercapacitors and their specimens were characterized to determine their electrochemical response including their specific capacitance and energy and power densities. These results were then compared with previous studies.

## 2. Materials and Methods

### 2.1. Materials and Sample Preparation

**Figure 1.**
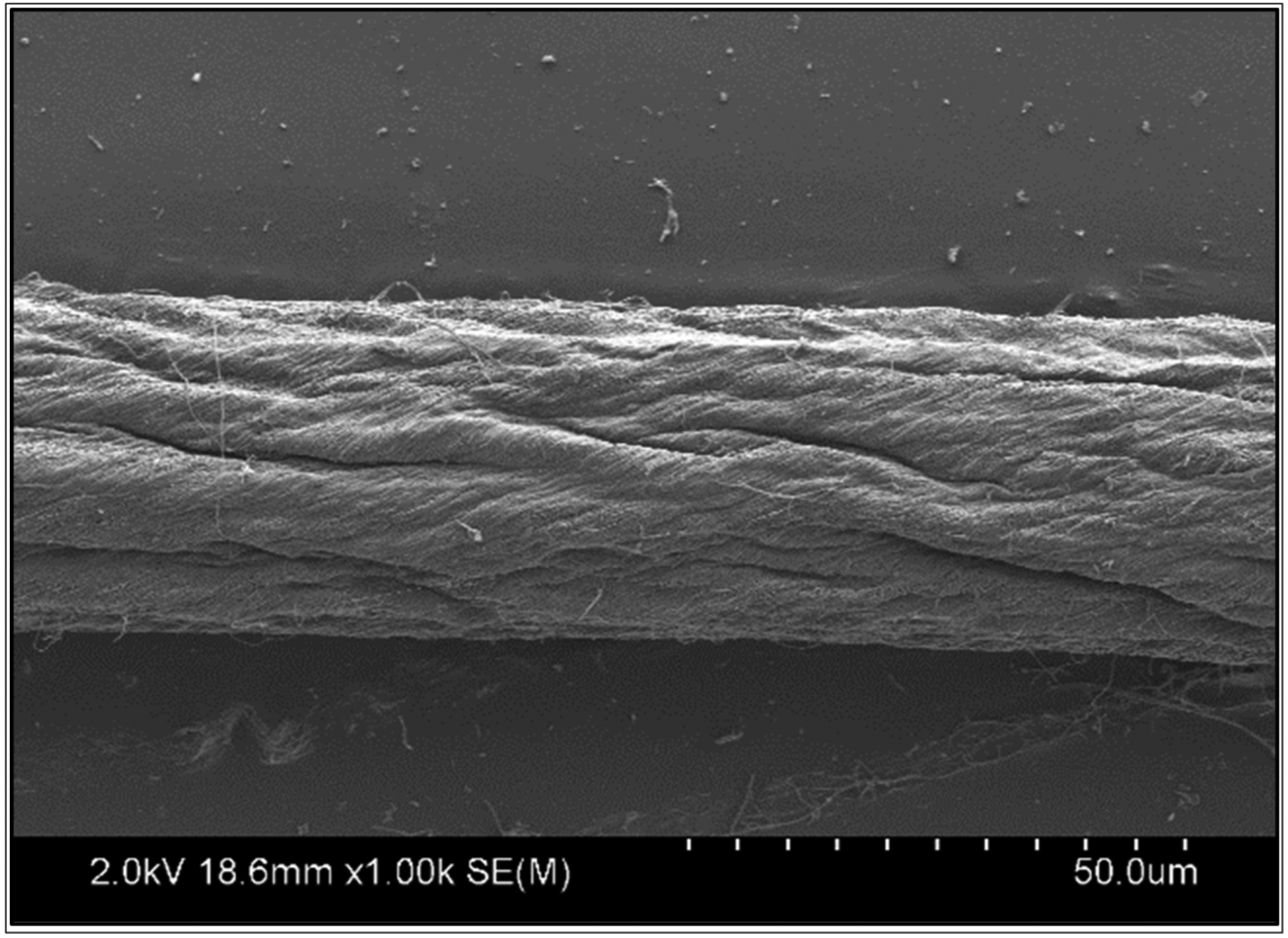
Scanning Electron Microscope (SEM) image of one-thread CNTY.

The CNTY used in this research was made from a vertically aligned carbon nanotube (CNT) array with no post-processing at Nanoworld Laboratories (University of Cincinnati, Cincinnati, OH, USA). The Si wafer, including a 5 nm alumina buffer layer and 1.2 nm catalyst, was laden into a chemical vapor deposition (CVD) reactor. The growth of CNTs on the catalyst sites of the Si wafer was achieved by heating the reactor to 750 °C in the presence of Ar and C_2_H_4_. The CNT array separation was achieved by contact with Ar and H_2_O during cooling. The CNT array, consisting of single-wall CNTs, was knit into yarn by dry spinning. The diameter, density, angle of twist, and average electrical resistivity of the densified CNTYs were ~30 μm, ~0.65 g/cm^3^, ~30°, and 1.7 × 10^−3^ Ω cm, respectively. The CNTYs over the graphene oxide (GO) sheet were initially distributed uniformly through the process of sonication in deionized water without ions. Following this, the uncured precursor of polydimethylsiloxane (PDMS) was incorporated into the graphene oxide dispersion. The combination was stirred constantly to guarantee an even allocation of graphene oxide within the polydimethylsiloxane. Utilizing the primed PDMS-GO gel electrolyte in tandem with the CNTY, progress was achieved in the construction of CNTY-based supercapacitors [26]. Figure 1 shows an image of the Scanning Electron Microscope (SEM) image of a one-thread CNTY.

### 2.2 Preparation of Polydimethylsiloxane (PDMS)

To prepare the polydimethylsiloxane (PDMS), the following steps were followed using its pre-polymer form acquired from a global lubricants supplier. It was sourced as a kit consisting of the PDMS base and a curing agent. The PDMS base and curing agent were in a predefined ratio, typically 10:1, in a clean glass container. This ratio was based on weight; for example, for every 10 g (A) of PDMS base, 1 g of the curing agent (B) was used. The PDMS base and curing agent mixture were then thoroughly mixed until a homogeneous solution was obtained. To eliminate any air bubbles trapped during the mixing process, the container was placed in a vacuum desiccator and subjected it to a process called degassing. During this, vacuum was applied until bubbles rising to the surface of the mixture were no longer observed. The degassed PDMS mixture was then poured into a mold of the desired shape for the supercapacitor application. It was then placed in an oven and cured at 70 °C for approximately 2 h. The curing process allowed the PDMS to transform from a liquid state to a flexible, solid state. After curing, the PDMS was allowed to cool to room temperature. Once cool, the now solid PDMS was carefully removed from the mold. At the end of this process, a solid, flexible piece of PDMS was ready to be utilized as the base for the PDMS–GO gel electrolyte in the fabrication of the CNTY-based supercapacitors [27].

### 2.3. Preparation of Graphene Oxide

Hummers’ method or similar oxidation processes are used to make graphene oxide from graphite. As a result, graphite expands and oxidizes, producing a compound called graphite oxide or graphite intercalation compound. Graphene oxide (GO) was prepared by cooling 46 mL of concentrated sulphuric acid until 7 °C. The sodium nitrate was gradually added at 0 °C with 0.0176 mol (1.5 g) in the following steps. In a mixture containing 2 g of prepared GO (prepared by calcining straw for 2 h at 500 °C for 2 h), potassium permanganate was constantly added over 15 min (0.042 mol, 6 g). After a 5 min ice bath, the mixture was magnetically stirred for two hours at 25 °C in the laboratory. Then, 46 mL of distilled water was added as drops for 20 min, followed by 98 °C for 20 min, then 140 mL of warm distilled water at 50 °C was added after a minute. After 10 min, the mixture was stirred with 15 mL hydrogen peroxide. The mixture was prepared for 30 min, then 300 mL of distilled water was added, and the mixture was kept without stirring for 24 h, then collected by filtering and kept drying at temperature (60–70 °C) until weight stabilized at 1.45 g [28–32].

### 2.4. Preparation Gel Electrolyte

To create an ion-transporting solution using the water-soluble polyvinyl alcohol (PVA) along with the lithium chloride (LiCl) salt, the following steps were taken: using a dissolved PVA solution in water (25 mL) at 85 °C, LiCl (0.075 mol) was added. The solution was transfererd into a 10 mL mold and sealed it. The mold was stored at −20 °C for 12 h, then thawed at room temperature for 12 h. A PVA/LiCl gel electrolyte was obtained after three freeze-thaw cycles [33].

### 2.5. Fabrication of CNTY-Based Supercapacitors

The production technique for CNTY-based energy storage devices may be summarized in this manner: initially, the CNTs must be spun into yarns using a dry spinning method. At that point, the CNTYs go through a twisting procedure to improve their strength and conductivity. Lastly, the twisted CNTYs are wound onto a spindle.

The formation of the supercapacitor commenced the moment the CNTY electrodes attained readiness. The architecture of the supercapacitor could manifest itself as spiraled or stratified, contingent on the coveted utilization and schematic stipulations. For a typical symmetrical dual-electrode arrangement, a duo of CNTY electrodes was wound around a partition (which could be PDMS–GO or PVA–LiCl gel electrolyte) to impede electrical conjunction between them. The partition also allows the flux of ions amid the electrodes during charging and discharging.

The supercapacitor was secured and enclosed after compilation, preventing the movement of the electrolyte, and ensuring physical durability. This result could be attained by employing an assortment of techniques, such as manipulating a thermally adhesive polymer or epoxy adhesive, contingent on the requested purpose and functioning environments.

#### 2.5.1. CNTYs Supercapacitor GO/HCL/CMM

**Figure 2.**
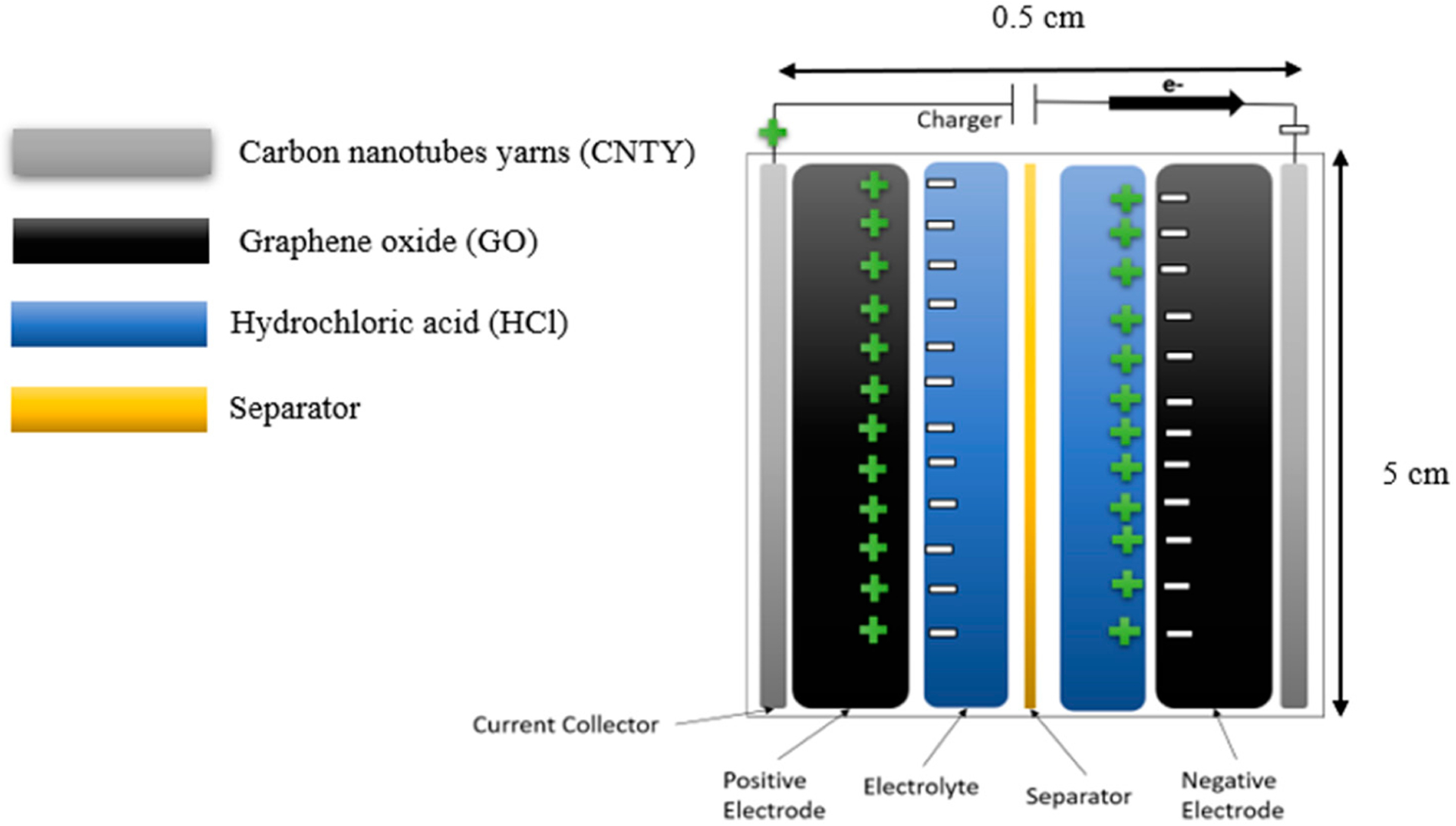
Schematic of sample and dimensions.

Figure 2 shows that the CNTY SC layers in an electrode of GO, hydrochloric acid (HCl), and Celgard^™^ 2400 Monolayer Membrane (CMM) from Celgard (Charlotte, NC, USA), as separate in their specific dimensions. When combined in the appropriate proportions, these components form an effective supercapacitor for storing energy [6].

#### 2.5.2. CNTY Supercapacitor GO/Zinc Sulfate-PVA/LiCl/PDMS

**Figure 3.**
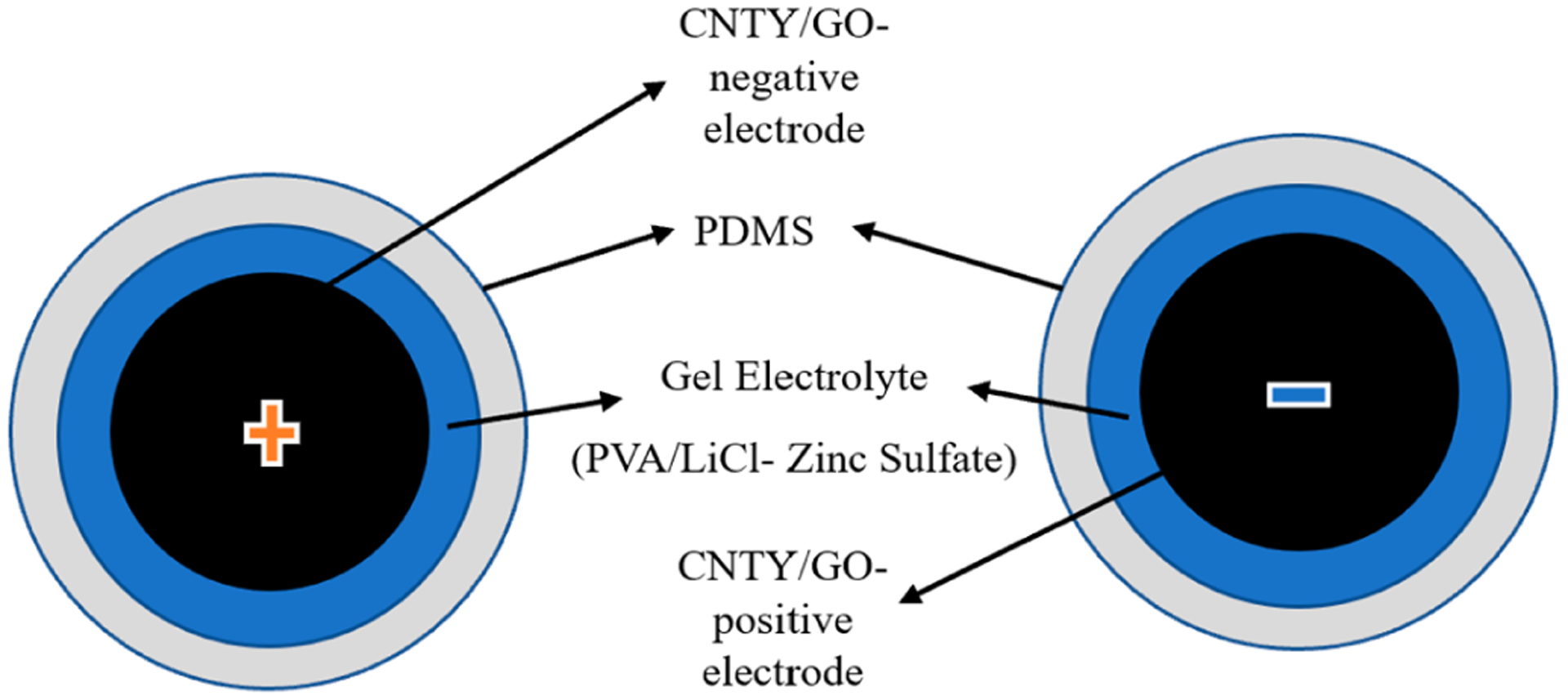
Schematic of GO/zinc sulfate-PVA/LiCl/PDMS/CNTY of flexible solid-state linear supercapacitor.

As part of the fabrication process for the second and third samples of the CNTY supercapacitor, the gel electrolyte consisted of GO/PVA/LiCl, and the CNTYs had to be immersed and coated in the gel and zinc sulfate, and then encased in PDMS to enhance structural and insulative properties. As soon as the final assembly had been cured, connected to an external circuit, and tested for supercapacitor performance, an external circuit was connected [34], as depicted in Figure 3.

### 2.6. Data Analysis and Interpretation

An electrochemical workstation (Bio-Logic SP-300) was used for all electrochemical characterization of single electrodes. The Bio-Logic SP-300 electrochemical workstation provides fast, sensitive, stable, and modular electrochemical characterization. Cyclic voltammetry (CV) tests were performed at various scan rates (30, 40, 50, 100, 150, and 200 mV/s). Galvanostatic charge–discharge (GCD) tests of the experiments were conducted using a potential range from 0 to 2.7 V at the current densities of 0.5, 1, 2, and 3 A/g.

The comprehensive analysis performed with this advanced electrochemical apparatus generated a wealth of data. The assessment of CNTY supercapacitors (CNTY SCs) involved determining their specific capacitance: gravimetric, volumetric, and areal (*C*_m,_ *C*_v_, and *C*_a_). Gravimetric capacitance can be calculated based on the galvanostatic charging/discharging curve described by Equation (1) [35].

(1) $$C_{\text{m}}=\frac{I\times \Delta t}{\Delta V\times m}$$

where *C*_m_ is the gravimetric capacitance, *I* is the current density, Δ*t* is the discharging time, Δ*V* is the potential window, and *m* is the mass of the electroactive material. Based on the values for *C*_m_ and volume of the electrode, the total capacitance of the electrodes can be calculated from Equation (2) [35].

(2) $$C_{\text{V}}=\frac{C_{\text{m}}\times \rho }{V}$$

where *C*_v_ is the volumetric capacitance, *V* is the total volume of the electrode, and *ρ* is its density. The areal capacitance can be calculated from *C*_v_ and the projection area of the electrode according to Equation (3) [35].

(3) $$C_{\text{a}}=\frac{C_{\text{V}}}{A}$$

where *C*_a_ is the areal capacitance and *A* is the surface area of the sample electrode.

## 3. Results and Discussion

### 3.1. CNTY GO/Zinc Sulfate/PDMS/SCs

The results of this study illustrate how well this GO/zinc sulfate/PDMS CNTY supercapacitor works. It demonstrates how much energy density can be stored, how quickly it can release that energy, and how many times this can be performed before it breaks down. The mixed number of current density and potential can be provided with the CV test and GCD test given the time it will take to change.

**Figure 4.**
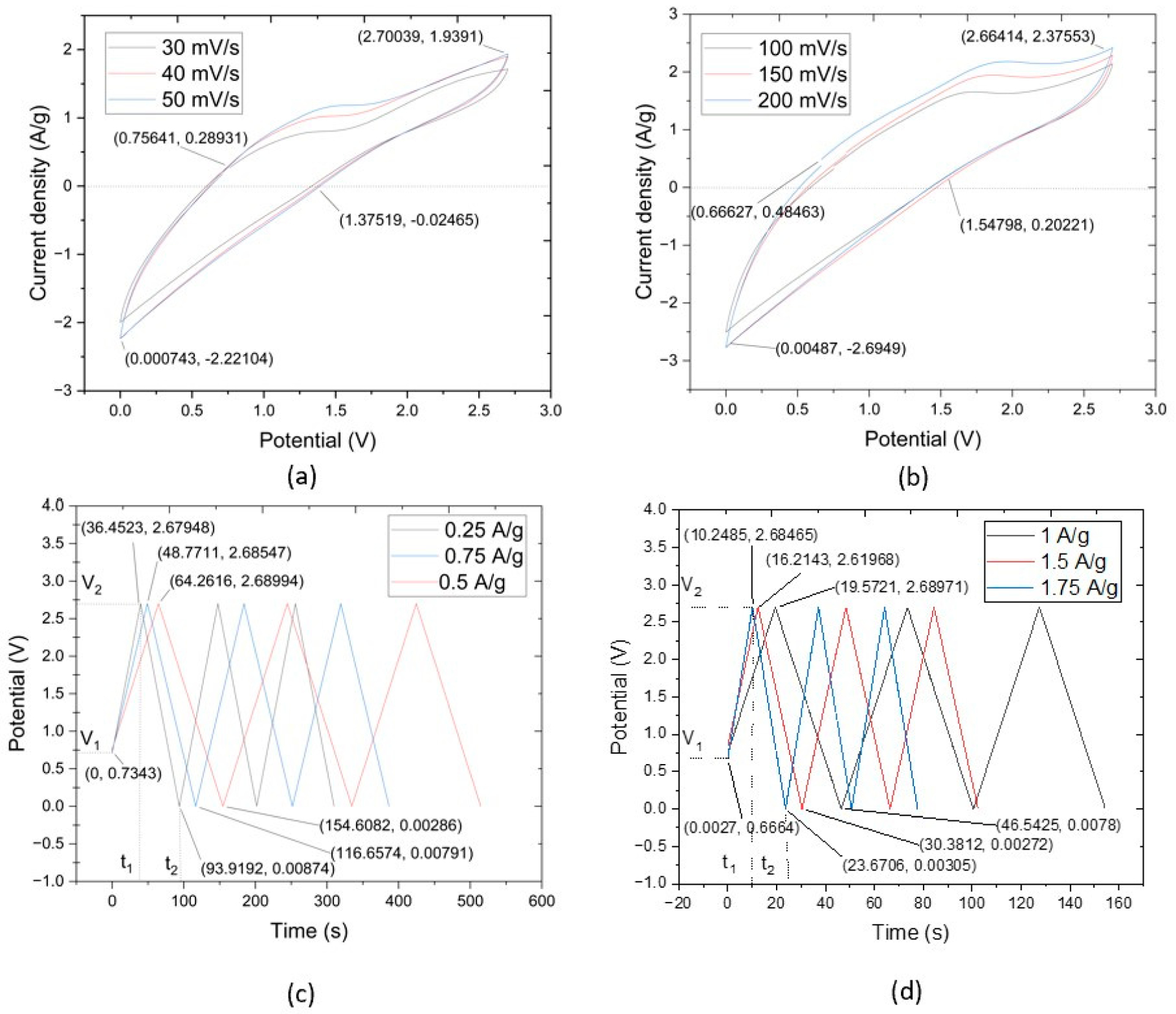
CV curves of CNTY GO/Zinc Sulfate/PDMS/SC samples with scanning rate range: (**a**) 30–50 mV/s, (**b**) 100–200 mV/s. GCD curves of CNTY GO/Zinc Sulfate/PDMS/SC at (**c**) 0.25–0.75 A/g, (**d**) 1–1.75 A/g.

The results of the CV test concern the utilization of CNTYs as electrode materials in supercapacitors. CNTYs, endowed with superior stability, cycle life, and performance, are ideal candidates for next-generation energy storage systems. Through a meticulous fabrication process that incorporates a thin layer of CNTY embedded with graphene, separator, PMDS, zinc sulfate, and other critical components, a sample was successfully produced. The sample was a flexible solid-state linear supercapacitor, and had a total mass of 54.25 g, as depicted in Figure 4a. The CV tests exhibited a current density of 0.624 A/g and a potential of 0.011 V. The maximum value of current density was 1.92 A/g, and the potential was 2.38 V, while the discharge values were recorded at 1.38 V for the potential and 0.013 A/g for the current density. CNTY’s stability and performance as a flexible electrode material were further assessed by evaluating the resistance change across different scan rates. The CNTY possesses considerable capacity to accumulate and dispel electric power, making it ideal for application in constructing high-achieving supercapacitors. This signifies that the CNTY can cache ample energy but can also quickly rejoin to fluctuations in power requirements, an attractive feature in supercapacitors.

The CV test results noted a current density of 0.756 A/g and a potential of 0.289 V. The values were lower than others because when the test became in the control of potential at 2.7 V, it was back to discharge mode. The discharge started when the current density dropped to 0 A/g.

The CV profiles obtained within the scan rate interval of 100 mV/s to 200 mV/s manifested as complete rings. This signified a pattern of capacitive reduction correlating to an increment in scan rates. Incorporating CNTY appeared to augment both the active and GO electrode interface mass. Detailed insights from the CV tests are captured in Figure 4a,b. The initial current density in Figure 4a was 0.756 A/g at the potential of 0.289 V. The maximum value of current density was 1.93 A/g at the potential of 2.70 V. The discharge values initiated from −0.024 A/g to 1.35 V. Similarly, Figure 4b started with a current density of 0.49 A/g and a potential of 0.67 V, escalating to a maximum current density of 2.39 A/g at a potential of 2.68 V. The CNTYs in this study had great capacitive behavior when compared with previous studies, as underscored by the largest current response. These characteristics may be attributed to the synergistic effect of the supercapacitors’ potential and current density.

The outcomes of the CV tests provide pertinent insights into the electrochemical processes of GO/zinc sulfate/PDMS/CNTY supercapacitors. The complete ring-shaped profiles, traditionally indicative of good capacitive behavior, aligned with a trend of decreasing capacitance with increasing scan rates, as observed in previous studies. Moreover, the integration of CNTY in the supercapacitor matrix seemingly enhanced both the active and GO electrode interface masses, contributing to the capacitive performance of storage energy. The differences and similarities between Figure 4a,b in terms of current density and potential values call for further investigations to unravel the underlying mechanisms and optimize the performance of these supercapacitors.

Figure 4c,d illustrate the galvanostatic charge–discharge (GCD) curves for the supercapacitors made of GO, zinc sulfate, PDMS, and CNTY over two separate current density ranges. Specifically, Figure 4c covers the range of 0.25 A/g, 0.5 A/g, and 0.75 A/g, while Figure 4d covers 1 A/g, 1.5 A/g, and 1.75 A/g. Both figures depict nearly linear charge curves for all current densities, paralleling the behavior of the corresponding discharge curves, an indication of excellent electrochemical performance in each sample.

The initial sudden voltage drop at the start of each discharge is attributable to the internal resistance of the GO electrode. Large voltage drops suggest a high internal resistance within the electrode and energy loss during the charge/discharge process. The maximum charge/discharge times observed in Figure 4c were 36.45, 48.77, and 64.26 s for current densities of 0.25 A/g, 0.5 A/g, and 0.75 A/g, respectively. In Figure 4d, these times extended to 10.24, 16.21, and 19.57 s for current densities of 1 A/g, 1.5 A/g, and 1.75 A/g, respectively.

The almost symmetrical and linear charge and discharge curves of the GO/zinc sulfate/PDMS/CNTY supercapacitors indicate exceptional electrochemical performance. These attributes are reminiscent of the desirable behavior reported in previous studies [29]. The presence of high internal resistance within the electrode, as reflected by large voltage drops, was observed during the charge/discharge process. These drops correlate to energy losses, which is a common challenge in supercapacitor design and merits further investigation. The swift cycling with each increase in current density likely results from the highly efficient ion and electron migration rates provided by the integrated CNTY layer in the supercapacitors. As the scan rates increased, a lower capacitance (572.62 F/g, 493.4 F/g, 426.4 F/g) and a distortion in the CV curves were noticed, potentially due to slower ion diffusion compared to electron transfer rates. The near-symmetrical profile shape, absence of abnormal peaks, and smooth potential window in both figures suggest that the CNTY and GO electrode have been properly oxidized. This promotes a steady charge/discharge rate throughout the CV cycle, which could potentially enhance the overall efficiency and lifespan of these supercapacitors.

### 3.2. CNTY GO/PVA-LiCl/PDMS/SCs

The second part of this study was centered on the analysis of the GO/PVA-LiCl/PDMS/CNTY supercapacitor. This analysis focused on key parameters such as energy density, power discharge rate, and durability through repeated cycles. The performance evaluation of these metrics was conducted using experimental procedures that included CV and GCD tests.

**Figure 5.**
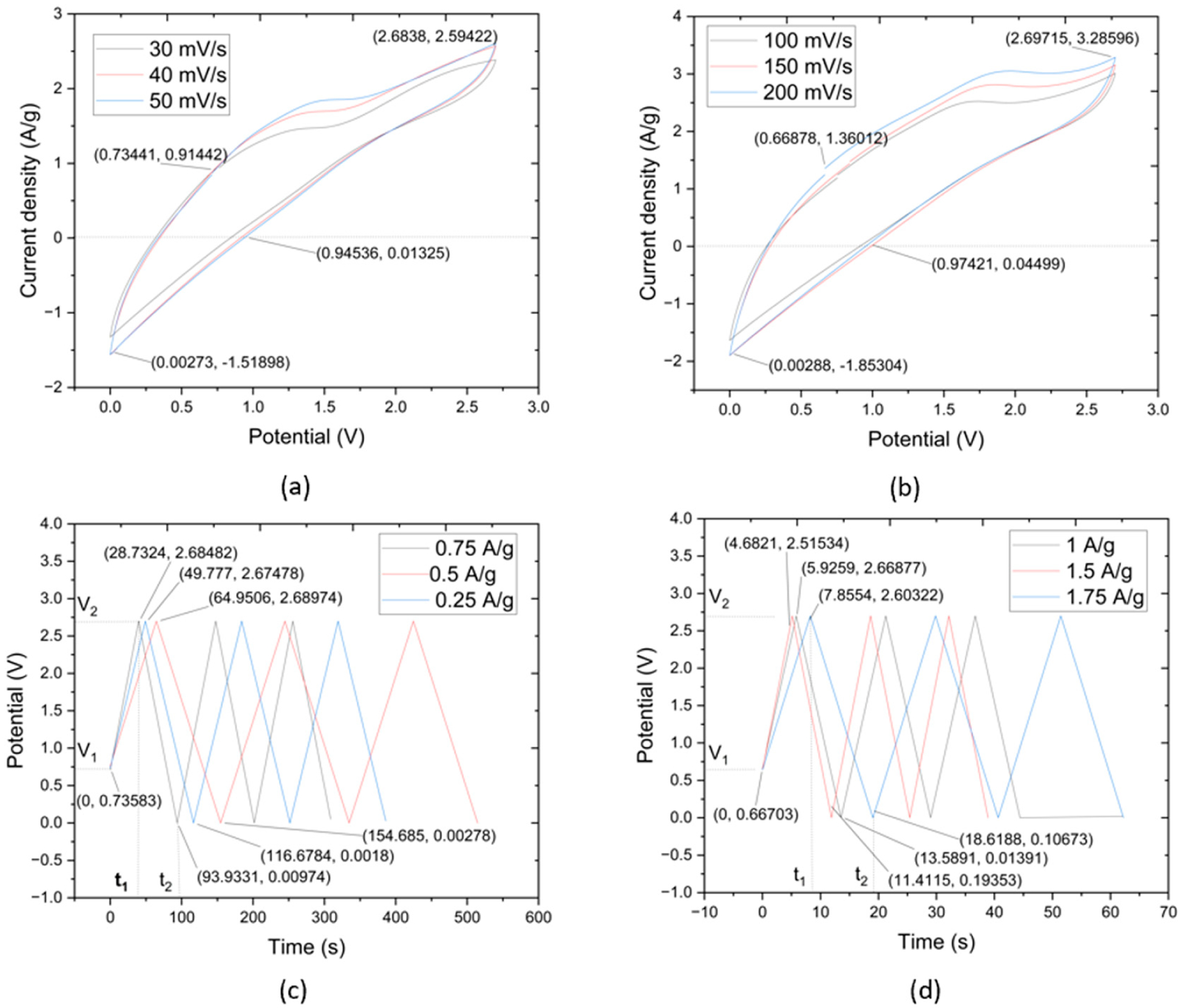
(**a**) CV curves of CNTY GO/PVA-LiCl/PDMS/SC samples with scanning rate range 30–50 mV/s, (**b**) CV curves of the samples with scanning rate range are 100–200 mV/s, (**c**) GCD curves of CNTY GO/PVA-LiCl/PDMS/SC at 0.25–0.75 A/g, (**d**) GCD curves at 1–1.75 A/g.

This research evaluated the suitability of CNTYs as electrode materials in supercapacitors, employing CV tests at a scan rate in the range of 30–50 mV/s. A flexible solid-state linear supercapacitor was carefully crafted, employing a technique that integrated a graphene-infused CNTY, PMDS, PVA-LiCl, and a separator, among other crucial components. The fabricated supercapacitor, which covered a total mass of 62.25 g, showcased excellent durability with no noticeable peeling, as depicted in Figure 5a. The performance and resilience of CNTY, acting as a flexible electrode material, were further substantiated by scrutinizing its resistance change across different scan rates.

The CV tests revealed a current density of 0.91 A/g and a potential of 0.73 V. The curves reached a maximum potential of 2.68 V and 2.59 A/g for current density, with discharge values of 0.94 V for potential and no value for current density. In contrast, Figure 5b showed the results of the CV test at a scan rate of 100–200 mV/s, which commenced with a current density of 1.36 A/g and a potential of 0.66 V. This escalated to a peak current density of 3.28 A/g at a potential of 2.7 V, with the discharge at 0.97 V for potential and 0.04 A/g for current density.

Based on the results illustrated in Figure 5c,d, supercapacitors made of GO/PVA-LiCl/PDMS/CNTY exhibit impressive electrochemical performance across a wide range of current densities. The initial voltage drop observed at the start of each discharge can be faster than the GO electrode’s internal resistance. In energy storage systems, internal resistance plays a crucial role—a high internal resistance can cause significant energy losses during the charge and discharge processes, leading to lower overall efficiency. In these results, this phenomenon is noticeable—large voltage drops, suggesting a higher internal resistance, indicate that some stored energy drops during the charge and discharge processes. It might be their mass where improvements could be made in future iterations of these supercapacitors. The charge/discharge times observed further underscore the performance of these supercapacitors. With times ranging from approximately 4.68 to 7.85 s at higher current densities of 1–1.75 A/g and from about 28.73 to 64.95 s at lower current densities (0.25–0.75 A/g), these supercapacitors demonstrate their capability to function efficiently across a wide range of operating conditions. These results underscore the promising electrochemical performance of the GO/PVA-LiCl/PDMS/CNTY supercapacitors. Such improvements could push the performance of these supercapacitors even further, making them a viable choice for various applications in the energy storage field. CNTYs demonstrated exceptional stability, long cycle life, and superior performance, leading to the proposition that they could be ideal candidates as electrode materials for cutting-edge energy storage systems.

### 3.3. CNTY GO/HCl/CMM/SCs

It is evident that the third phase of this study, which centered on the GO/HCl/CMM/CNTY supercapacitor, yielded the most promising results. The results from the third phase of this research contribute significant insights into the utilization of CNTYs as electrode materials for supercapacitors.

**Figure 6.**
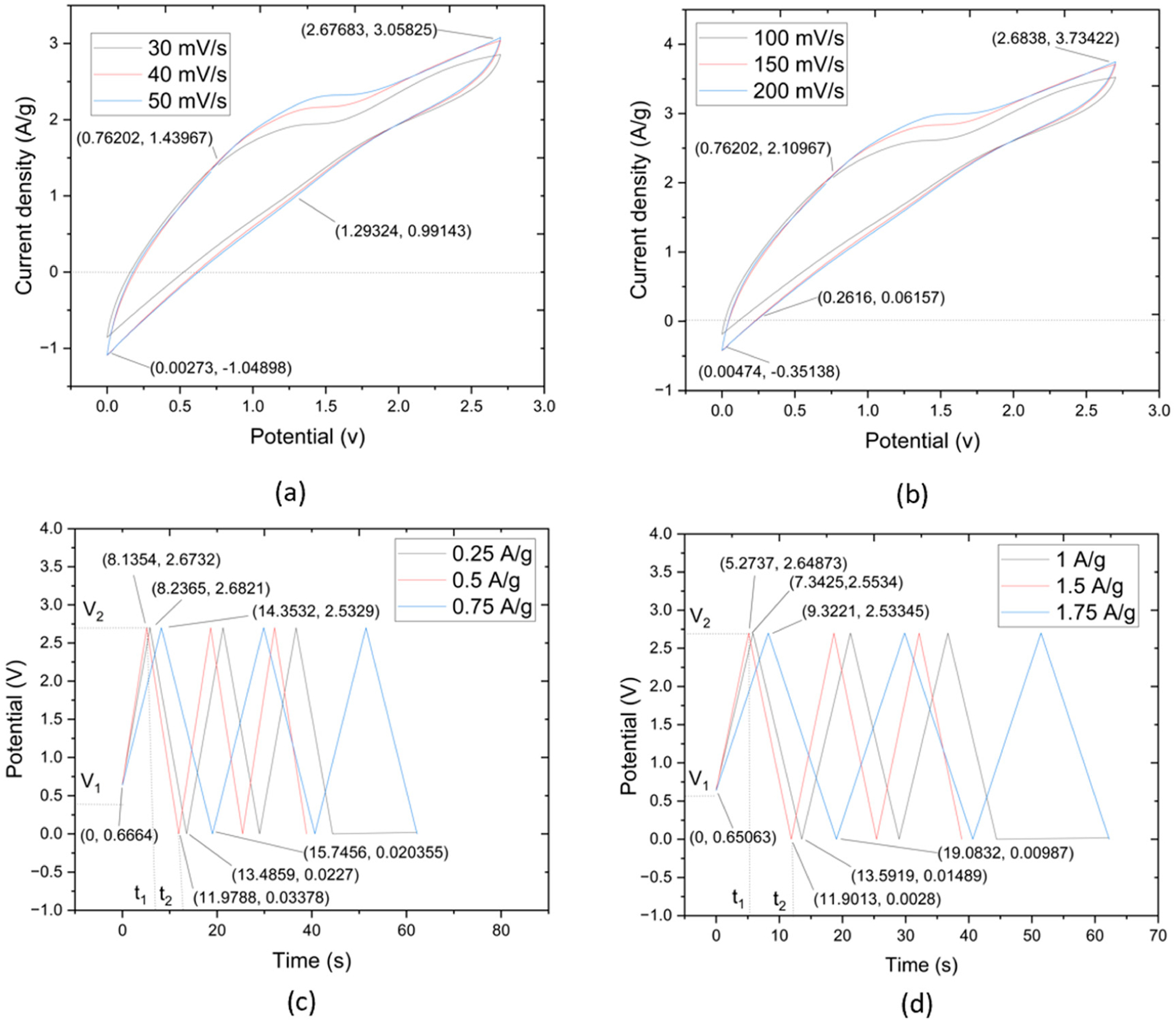
(**a**) CV curves of CNTY GO/HCl/CMM/SC samples with a scanning rate range of 30–50 mV/s, (**b**) CV curves of the samples with a scanning rate range of 100–200 mV/s, (**c**) GCD curves of CNTY GO/HCl/CMM/SC at 0.25–0.75 A/g, (**d**) GCD curves at 1–1.75 A/g.

The meticulously constructed supercapacitor, incorporating graphene-infused CNTY, GO/HCl/CMM, and a separator, among other key elements, showcased remarkable resilience and no noticeable deterioration. The supercapacitor’s total mass was 87.57 g, and, as depicted in Figure 6a, it showed no signs of peeling. Such results not only emphasize the durability of CNTYs when employed as flexible electrode materials but also highlight their ability to maintain consistent performance across varying scan rates.

The CV tests conducted during this phase of research further illustrated the superior performance of the GO/HCl/CMM/CNTY supercapacitor. The current density and potential of 1.43 A/g and 0.76 V, respectively, indicate robust energy storage and power discharge capabilities. The curves reaching their maximum at 2.67 V for potential and 3.05 A/g for current density underscore the supercapacitor’s ability to operate at high energy and power densities. Furthermore, discharge values of 1.29 V for potential and 0.021 A/g for current density demonstrate the supercapacitor’s ability to retain and release stored energy effectively.

The CV tests performed at a scan rate of 100–200 mV/s provided a more nuanced understanding of the supercapacitor’s performance under different operating conditions. Starting with a current density of 2.11 A/g and a potential of 0.76 V, and eventually reaching a maximum current density of 3.73 A/g at a potential of 2.68 V, the supercapacitor displayed its ability to operate efficiently under higher scan rates. The discharge values at 0.26 V for potential and 0.001 A/g for current density further validate this observation.

The results captured in Figure 6c,d demonstrate the impressive electrochemical performance of the GO/HCl/CMM/CNTY supercapacitors over a wide array of current densities. The curves in these figures, almost linear and running parallel for both charging and discharging, indicate an efficient energy storage and release process. This suggests that the supercapacitors have an excellent capacity for storing charge and can also release it effectively.

One point to note is the sudden drop in voltage at the onset of each discharge. It can be tied back to the internal resistance of the GO electrode. The marked voltage drops hint at a higher internal resistance, leading to energy losses during the charge and discharge cycles. The mass could benefit from some enhancements in future design iterations of these supercapacitors. The charge and discharge times further emphasize the strong performance of the supercapacitors. They ranged from roughly 8.13 to 14.35 s at higher current densities of 0.25 to 0.75 A/g and from around 5.27 to 9.32 s at the same current densities of 1 to 1.75 A/g. This is a testament to their ability to work efficiently across varied operating conditions. The results indicate promising electrochemical performance by the GO/HCl/CMM/CNTY supercapacitors.

### 3.4. Capacitance, Energy, and Power Densities

**Figure 7.**
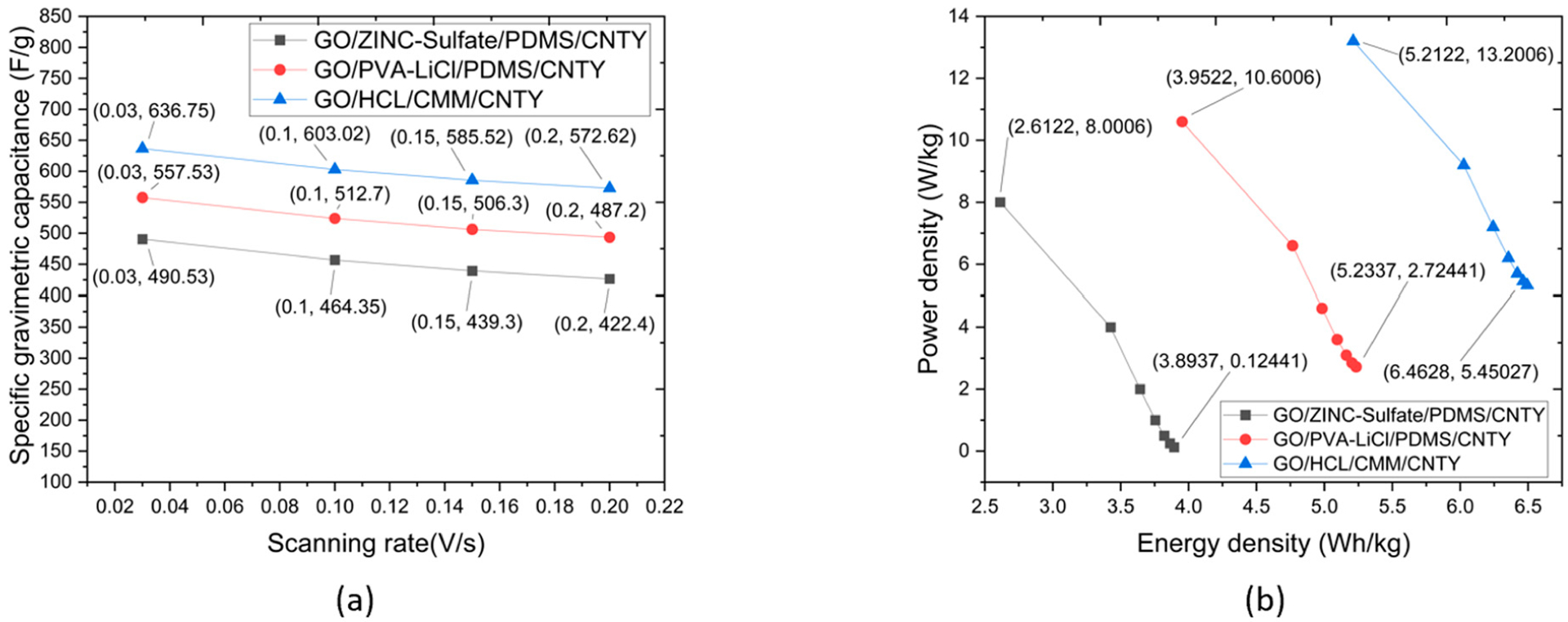
(**a**) Capacitance relation to the scanning rate for the GO/zinc sulfate/PDMS/CNTY, GO/PVA-LiCl/PDMS/CNTY, and GO/HCl/CMM/CNTY supercapacitors, evaluated at a range of 0.03 V/s to 0.2 V/s, (**b**) Ragone plot depicting the energy and power performance of the GO/zinc sulfate/PDMS/CNTY, GO/PVA-LiCl/PDMS/CNTY, and GO/HCl/CMM/CNTY supercapacitors.

A series of cycling tests were performed on the CNTY SCs across 12 cycles at varying current density ranges from 0.25 A/g to 1.75 A/g, and 2.25 A/g. The process was repeated four times. The results, depicted in Figure 7a, show a reduction in capacitance across all tested CNTY SCs when scan rates decreased from 0.03 to 0.2 V/s. Specifically, the supercapacitors made of graphene oxide (GO) with zinc sulfate, polyvinyl alcohol (PVA)-lithium chloride (LiCl), and hydrochloric acid (HCl)/Celgard^™^ 2400 Monolayer Membrane (CMM) along with CNTY showed a decrease in specific capacitance values from 490.53 to 426.4 F/g, 557.53 to 493.4 F/g, and 636.75 to 572.62 F/g, respectively. The reason for the decrease in the capacitance is that the power density has dropped off its charge.

During the cycling tests, all the CNTY SC samples showed an initial decrease in capacitance of 33.73 F/g, 44.83 F/g, and 26.18 F/g. When the scan rate was increased to 0.10 V/s, there was a further decrease of 17.5 F/g, 6.4 F/g, and 25 F/g. Interestingly, the decrease in capacitance was less, only 12.9 F/g, 19.1 F/g, and 16.9 F/g, when the scan rate was increased to 0.15 V/s. This outcome suggests that at higher scan rates, ions within the electrolyte of each sample have less time to penetrate the electrode material. As a result, they accumulate on the surface of the electrode, which then results in a decrease in capacitance. Such behavior is not unique to these CNTY SCs. Prior studies have also noted similar trends of decreasing capacitance at higher scan rates, which supports this study’s findings and reinforces the potential of CNTY SCs in advanced energy storage applications [29]. The Ragone plots of Figure 7b for the three results of CNTY SCs—GO/zinc sulfate/PDMS/CNTY, GO/PVA-LiCl/PDMS/CNTY, and GO/HCl/CMM/CNTY show-case these calculated values, derived from their respective galvanostatic charge–discharge (GCD) profiles at different current densities. The GO/zinc sulfate/PDMS/CNTY SC displayed mass energy density ranging from 2.6 to 3.9 Wh/kg and mass power density between 8.0 and 0.12 W/kg. For the GO/PVA-LiCl/PDMS/CNTY SC, the specific energy density varied from 4.0 to 5.2 Wh/kg, and the power density ranged from 10.6 to 2.7 W/kg. The GO/HCl/CMM/CNTY SC showed an energy density between 5.2 and 6.7 Wh/kg and a power density from 13.20 to 6.46 W/kg. Table 1 provides a comparison with materials used in previous studies.

In general, CNTY SCs exhibit higher power and energy characteristics compared to conventional electrochemical capacitors. In the present study, the Ragone plots for each SC sample showed a trend of increasing energy density coupled with decreasing power density. The resistance offered by the GO electrode incorporated into all samples leads to conclude thatis likely responsible for the observed decrease in power density. By reducing power density, the CNTY SC samples avoid rapid energy consumption, fostering a slower energy usage rate. As a result, these SCs enhanced with CNTY provide a more readily available energy source compared to batteries and standard capacitors. This study s used different materials to fabricate the CNTY SCs. The CV test results showed significant improvements compared to previous studies. The supercapacitors demonstrated a higher capacitance of 636.75 F/g compared to previous studies, such as [36], which reported a value of 263.31 F/g, and [42], which reported a value of 255.5 F/g [42]. This indicates that GO/HCl/CMM/CNTY possesses a higher capacity per unit mass for storing electrical charges. Regarding energy density, this study produced a result of 5.2 Wh/kg. The result was superior to the energy densities reported in [39–42]. It suggests that the supercapacitor in this study can store more energy per unit mass, making it a more efficient device. Moreover, the study observed a higher power density of 13.2 W/kg. The test was a remarkable improvement over the values reported in [42]. The supercapacitor in this study can deliver or absorb energy faster, making it highly desirable for applications that require rapid energy discharge. The material offers considerable advantages over the materials used in previous studies. These findings demonstrate the potential of the GO/zinc sulfate/PDMS/CNTY, GO/PVA-LiCl/PDMS/CNTY, and GO/HCl/CMM/CNTY in developing high-performance supercapacitors.

## 4. Conclusions

Supercapacitors using CNTYs embedded in various electrochemical-active matrices were fabricated and characterized to determine the capacitive properties, energy, and power densities. The results showed that the CNTY supercapacitors displayed excellent capacitive behavior, with nearly rectangular CV curves across a range of scan rates. The energy density and power density of these supercapacitors fluctuated between a minimum of 3.89 Wh/kg and 8 W/kg while the maximum was between 6.46 Wh/kg and 13.20 W/kg. The specific capacitance of these CNTY supercapacitors was significantly high, which further supports their potential for energy-dense applications. A significantly high capacitance, peaking at 636.75 F/g, evidence of the promise of such supercapacitors for energy-dense functions, was determined. This study underscores the potential of CNTY-based supercapacitors as high-performance energy storage devices. This overarching goal of the project aims to develop CNTY supercapacitors that can be integrated into polymeric and composite materials to power CNTY sensors that can measure strain and temperature or detect damage, among many others.

## Figures and Tables

**Table 1.** Comparison of relevant properties or various materials.

Study	Material	Potential (V)	Capacitance	Energy Density	Power Density	References
CNTY SC	GO-zinc sulfate-PDMS	0.0–2.7	490.53 F/g at 1.75 A/g	3.9 {Wh/kg]	8 [W/kg]	
CNTY SC	GO-PVA-LiCl-PDMS	0.0–2.7	557.53 F/g at 1.75 A/g	5.2 [Wh/kg]	10.6 [W/kg}	
CNTY SC	GO-HCl-CMM	0.0–2.7	636.75 F/g at 1.75 A/g	6.7 [Wh/kg]	13.20 [W/kg]	
MnO_2_@MWCNTs SSLSc	PVA-LiCl	0.0–1.0	8.5 F/cm^3^ at 1 A/cm^3^	0.96 [Wh/cm^3^]	2.5 [W/cm^3^]	[36]
Elastic SC based on CNTs sheet + rubber fiber	PVA-H_3_PO_4_	0.0–0.8	19.2 [F/g] at 0.1 A/g	0.363 [Wh/kg]	421 [W/kg]	[37]
CNTs/SS core–sheath SC	PVDF-HFP-EMIMBF_4_ (IL)	0.0–2.7	263.31 [F/cm^3^] at 0.1 V/s	66.7 [mW h/cm^3^]	8.89 [W/cm^3^]	[38]
Pt filament-based CNTs symmetric SC	PVA-H_3_PO_4_	0.0–1.0	241.3 [μF/cm] at 5 mV/s 86.2 [F/g] at 5 mV/s	35.27 Wh/kg	10.69 kW/kg	[39]
SWCNTs yarn SC	PVA-H_3_PO_4_	0.0–1.0	5 F/g	0.6 Wh/kg	-	[40]
Electrochromic SC based on CNT/PANI + Elastic rubber FSSC	PVA-H_3_PO_4_	0.0–1.0	255.5 [F/g] at 1 A/g	12.75 [Wh/kg]	1494 [W/kg]	[41]
Strip-shape CNT/PANI SC	PVA-H_3_PO_4_	0.0–1.0	421.7 [F/cm^3^] at 0.5 A/cm	9.6 [mWh/cm^3^]	2.91 [W/cm^3^]	[42]

## Data Availability

The data have been obtained from the experimental results.
